# Development and Validation of the Compensatory Belief Scale for the Internet Instant Gratification Behavior

**DOI:** 10.1016/j.heliyon.2024.e23972

**Published:** 2024-01-02

**Authors:** Bin Yin, Yong Shen

**Affiliations:** a Laboratory for Learning and Behavioral Sciences, School of Psychology, Fujian Normal University, Fuzhou, 350117, Fujian, China; b Department of Applied Psychology, School of Psychology, Fujian Normal University, 350117, Fujian, China

**Keywords:** Internet Instant Gratification Behavior, Compensatory belief, Compensatory Belief Scale for the Internet Instant Gratification Behavior (CBS-IIGB), Delayed gratification, Internet temptation

## Abstract

**Objective:**

The concept of “compensatory belief” pertains to an individual's conviction that the negative consequences of a specific behavior can be counteracted by engaging in a subsequent positive behavior. This study sought to devise a scale tailored to gauge compensatory beliefs concerning internet instant gratification behaviors.

**Methods:**

Utilizing the Credamo online questionnaire platform, data were amassed from two distinctive cohorts: organizational employees and college students. A collective 1064 responses were amassed.

**Results:**

The newly created Compensatory Belief Scale for Internet Instant Gratification Behavior was bifurcated into two versions: one tailored for employees (CBS-IIGB-E) and the other for college students (CBS-IIGB-S). Through an exploratory factor analysis, two factors were discerned, namely “Compensatory Beliefs for Working/Studying " and “Compensatory Beliefs for Resting”. A confirmatory factor analysis validated this two-factor model with the following metrics for the employee version: SBχ2 = 54.88, *df* = 32, CFI = 0.974, TLI = 0.964, RMSEA = 0.064, SRMR = 0.047, and for the student version: SBχ2 = 19.26，*df* = 19，CFI = 0.999，TLI = 0.999，RMSEA = 0.008，SRMR = 0.033. The scores on the Internet Addiction Scale and the Smartphone Addiction Scale showed a positive correlation with the overall scores of the CBS-IIGB-E and the scores of its two factors. Conversely, the scores on the Self-control Scale exhibited a negative correlation with the total scores of the CBS-IIGB-E and the scores of its two factors. The correlation pattern with the criterion scales for the CBS-IIGB-S mirrored that of the CBS-IIGB-E, with the exception that the Self-control Scale only correlated with the two factors and not the overall score of the CBS-IIGB-S. The McDonald's Omega coefficients for the two factors of the CBS-IIGB-E were 0.84 and 0.86 respectively, and those for the CBS-IIGB-S were 0.82 and 0.87 respectively. Both scales demonstrated a test-retest reliability of 0.76. Significant differences in the scale scores across diverse target groups were observed in the simulation game of internet instant gratification behavior, thereby validating the predictive validity of the developed scales.

**Conclusion:**

The Compensatory Belief Scale for Internet Instant Gratification Behavior (CBS-IIGB) is a reliable and valid tool for measuring compensatory belief in situations where the allure of immediate internet gratification comes into conflict with long-term objectives, among both organizational employees (CBS-IIGB-E) and college students (CBS-IIGB-S).

## 1 Introduction

The digital era has ushered in a widespread consumption of internet-based entertainment, offering users instant satisfaction, customized content, and delightful experiences at minimal exertion. This trend, known as “instant gratification”, stands in contrast to “delayed gratification”, both constituting “intertemporal choice” — a decision-making process balancing immediate pleasures and future benefits [1]. Current research underscores an increasing preference for instant gratification, especially via digital channels such as online gaming, short video platforms, or online streaming [2,3]. Defined as the quest for immediate pleasure through online avenues, Internet Instant Gratification Behavior (IIGB) is characterized by its ease, often a mere device click away. This effortless accessibility, combined with the attraction of prompt rewards, propels us to examine the cognitive dynamics that dictate the equilibrium between yielding to immediate pleasures and staying committed to long-term objectives.

Psychology's rich tapestry of theories and models has frequently delved into the delicate balance between present desires and future aspirations. While the Theory of Planned Behavior [4] and the Health Belief Model [5,6] have been instrumental in this endeavor, the Compensatory Health Beliefs (CHB) model offers a distinct lens. Stemming from the works of Rabia and colleagues [[7], [8], [9]], this model elucidates individuals' intrinsic belief system, suggesting that the detrimental effects of one action can be counteracted by a subsequent beneficial behavior. A classic example is the rationalization of indulging in a calorie-laden dessert with a compensatory promise of an extended workout session later. Such beliefs, especially pronounced among dieters, offer a cognitive bridge to justify immediate temptations [10,11]. The CHB model posits that when individuals are pulled between temptations and enduring goals, they encounter cognitive dissonance [12] — a mental discomfort stemming from conflicting beliefs or values. In such situations, individuals might either avert the temptation, embrace it, or activate their compensatory beliefs (CBs). Given the cognitive comfort of the last approach, many lean towards CBs, aligning their immediate whims with their overarching objectives [13]. Yet, the journey does not end here; many falter in actualizing these compensatory actions, leading to remorse and waning self-confidence. The CB theory accentuates the pivotal role of post-decision self-efficacy. Those with inherent low self-assuredness may neglect the actual implementation of the compensatory action, risking a descent into detrimental cycles, including potential Internet addiction. In contrast, successful realization of compensatory actions can bolster self-efficacy, propelling individuals to break free from these detrimental loops and adopt healthier behavioral patterns.

**Fig. 1:**
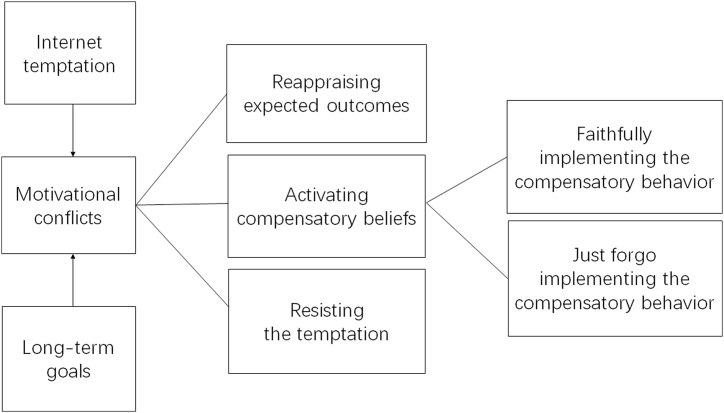
The Compensatory Belief Model for Internet Instant Gratification Behavior (Authorized reuse from Ref. [16]).

The versatility of the CB theory is evident in its application across diverse domains from health behaviors to driving safety and environmental considerations [8,[13], [14], [15]]. Building upon this foundation, our prior research substantiates the utility of the CB theory in understanding the cognitive processes steering individuals when enticed by online allurements and the subsequent psychological and behavioral repercussions [16]. A model based on the CB theory for Internet gratification behaviors is illustrated in Fig. 1.

While much of the existing discourse on IIGB orbits around internet addiction, equated with pathological internet usage [17,18], there's an expansive non-pathological segment that merits scrutiny. From a preventive healthcare perspective, discerning the behaviors and compensatory beliefs of this broader cohort is of paramount importance. Numerous studies converge on the idea that the essence of both substance and behavioral addictions is rooted in the quest for instant gratification paired with dwindling self-control [[19], [20], [21]]. Thus, unchecked indulgences, be it for escapism or relaxation, can inadvertently set the stage for internet addiction [22]. Furthermore, an unchecked dive into constant instant gratifications might not be synonymous with well-being. More often, it spirals into negative psychological states, characterized by heightened stress, regret, guilt, anxiety, and compromised sleep [[23], [24], [25], [26]]. These adverse psychological aftermaths, coupled with the triggering events, serve as harbingers of depressive symptoms [27].

In light of this, gauging attitudes towards CBs emerges as a pivotal precursor to understanding IIGB. While traditional methods, such as questionnaires and interviews, have been pivotal [14,15,[28], [29], [30], [31], [32]], the budding nature of CBs in the IIGB context necessitates rigorous validation. Moreover, the often-latent long-term goals that IIGB might eclipse typically unfurl over extended periods, emphasizing the imperative for longitudinal research. A foundational step in this direction is the crafting of reliable measurement instruments.

A nuanced perspective is essential when navigating the dichotomy of immediate versus delayed gratification, as the behaviors of college students and organizational employees differ, shaped by their developmental milestones and life circumstances. Weekends, for instance, signify different things for these groups. While academic structures grant students significant autonomous learning leeway, blurring weekday-weekend distinctions, employees, tethered to work obligations, usually find respite only during weekends. Such lifestyle variations can shape distinct attitudes towards time, potentially influencing the efficacy of standardized questionnaire measures. Moreover, diverging definitions of academic and occupational delayed gratifications [33,34] spotlight the unique decisions each group faces: students juggle immediate pleasures that might impede academic pursuits against long-term academic aspirations, whereas employees weigh immediate relaxation against long-term career growth. Recognizing these nuanced differences underlines the need for tailored scales to accurately gauge CBs-IIGB across these distinct demographics.

Bridging this gap, our study, rooted in the CHB theory, aspires to craft the Compensatory Belief Scale for Internet Instant Gratification Behavior (CBS-IIGB), bifurcated into versions for employees (CBS-IIGB-E) and students (CBS-IIGB-S). This segmentation ensures the tailored measurement needs of distinct groups are met, and these scales are scrutinized for item discrimination, structural dimensions, and construct validity. We define “CBs-IIGB” as the degree to which an individual believes they can neutralize the adverse effects of internet instant gratification through compensatory actions. Elevated scale scores signify robust CBs and an augmented propensity to succumb to instant gratifications. These scales hold promise for mental health assessments during college admissions or job onboarding, aiming to discern group variations in response to online temptations. This proactive approach facilitates the early identification of vulnerable individuals, paving the way for timely, adaptive interventions rooted in preventive care. This endeavor seeks to thwart potential mental health challenges stemming from internet addiction or a dearth of compensatory behaviors, amplifying the work and academic efficacy of evaluated individuals while championing mental well-being.

## 2 Materials and methods

### 2.1 Participants

The study utilized the precision delivery function on the online smart research platform *Credamo*^1^ to disseminate the survey nationwide. The chosen sample sizes for each phase of the study were based on feasibility considerations and the iterative nature of scale development. Additionally, given the exploratory nature of this research and the lack of prior data on this specific topic, these sample sizes were deemed adequate for initial scale validation. In the first preliminary test, college students were selected as the sample and 219 responses were collected. After eliminating participants who did not answer seriously based on screening questions (e.g., “This question tests whether you are answering seriously. Please select ‘Strongly Disagree'."), 200 valid questionnaires were adopted, consisting of 105 males and 95 females, with the grades ranging from freshman to graduate students. The second preliminary test involved the release of two versions of the questionnaire, one for employees and the other for students. The student version received 328 questionnaires, of which 282 valid ones were adopted after screening, including 98 males and 184 females. The employee version received 226 questionnaires, with 187 valid ones adopted after screening, including 105 males and 82 females. The third round was the official test, where the employee version collected 219 questionnaires and the student version collected 226. After screening, 195 valid questionnaires were adopted for the employee version (97 males and 98 females) and 200 for the student version (63 males and 137 females). The demographic information of the test samples is presented in Table 1.

**Table 1:** Basic demographic information of test samples.

Student Sample				Employee Sample			
First Test	Age	18–22	68.09 %	Second Test	Age	≤20	1.94 %
		23–26	26.60 %			21–30	51.46 %
		27–30	3.19 %			31–40	33.01 %
		>30	2.13 %			41–50	9.71 %
	Year of Study	Freshman	1.06 %			>50	3.88 %
		Sophomore	24.47 %		Educational Background	High school or below	9.71 %
		Junior	39.36 %			Associate degree	11.65 %
		Senior	20.21 %			Bachelor's degree	69.90 %
		Graduate or above	14.89 %			Graduate degree	8.74 %
					Work Experience	Less than 3 years	17.48 %
						3–5 years	27.18 %
Second Test	Age	18–22	78.36 %			6–10 years	36.89 %
		23–26	20.15 %			11 years or above	18.45 %
		27–30	1.49 %				
		Freshman	6.72 %				
	Year of Study	Sophomore	28.36 %				
		Junior	26.12 %	Third Test	Age	≤20	0.50 %
		Senior	21.64 %			21–30	51.50 %
		Graduate and above	17.16 %			31–40	39.00 %
						41–50	7.00 %
						>50	2.00 %
Third Test	Age	18–22	85.94 %		Educational Background	High school or below	6.00 %
		23–26	13.54 %			Associate degree	14.50 %
		27–29	0.52 %			Bachelor's degree	68.50 %
	Year of Study	Freshman	23.96 %			Graduate degree	11.00 %
		Sophomore	23.44 %		Work Experience	Less than 3 years	22.50 %
		Junior	26.04 %			3–5 years	27.00 %
		Senior	14.06 %			6–10 years	31.50 %
		Graduate or above	12.50 %			11 years or above	19.00 %

Two weeks later, the sample collection function of *Credamo* was used to re-issue questionnaires to the aforementioned official test samples to measure test-retest reliability. The employee version collected 104 responses, and the student version collected 91. After eliminating participants who did not answer seriously through screening questions, 100 valid questionnaires were adopted for the employee version and 86 for the student version.

## 3 Scale development

### 3.1 Theoretical construction

In the formative stage of our research, we adhered to the principle of theoretical saturation, conducting in-depth interviews with a strategically selected sample of 10 participants. This sample consisted of a balanced gender distribution and was composed of both university students and working professionals. We employed a grounded theory approach to analyze the interview data, focusing on the participants' experiences with online temptations and long-term goals. Interview questions were crafted to elicit rich narrative data and included queries such as: “What is your most frequent form of online entertainment?” “Do you have long-term goals for personal development? How do you typically choose when these goals conflict with your online entertainment habits?” “If you opt for entertainment first, what kind of compensation do you usually make for your personal goals?” “Do you usually carry out these compensatory actions? How long after entertainment do you make compensation?” “What feelings do you experience if you cannot carry out compensatory actions afterwards?”. Based on the interview content, we explored participants' choices and the methods of compensation they adopt when they face a conflict between online temptations and long-term goals.

Through thematic analysis, we identified three key sources of internet instant gratification temptation — online games, online drama, and online shopping — as well as three prevalent decision-making strategies: resisting temptation, yielding to temptation, and activating CBs. After activating CBs, they would either carry out or not carry out compensatory behaviors (Fig. 1). These qualitative insights informed the creation of an Interactive-Narrative-Style Game-Based Assessment (IN-GBA) to generate a more nuanced behavioral dataset [16]. This tool was designed to emulate real-world choices in internet instant gratification scenarios, thereby enriching the data necessary for scale development.

### 3.2 IN-GBA development and purpose

The IN-GBA was conceptualized to provide a tangible platform to test the CB theory in the IIGB context. Participants are presented with three distinct scenarios — online gaming, online shopping, and online drama — to choose from, simulating real-world conflicts between immediate gratification and long-term objectives. Those unfamiliar with all three scenarios were excluded from the target population. Each scenario simulated the conflict between personal long-term goals and immediate gratification from online temptations, establishing five logically consecutive temptation items. The participant could choose to resist the temptation with resoluteness, cognitively reappraised expected outcomes and accepted the temptation, or activate their CBs before accepting the temptation. If a participant chose to resist the temptation, another tempting situation would appear in the next question, where the participant could continue making one of the three choices until they rejected all five temptation items or chose to accept the temptation after activating their CBs on a certain item. If a participant chose to activate their CBs in any of the five items, the questionnaire would redirect them to a scenario testing if they would carry out compensatory behaviors. When the scenario items were completed, the assessment would display a story ending corresponding to the participant's various choices and ask if they regretted their choices, how they felt about them, and any other comments they might have after the scenario simulation.

The primary objectives of this IN-GBA were two-fold: 1) to delve into the application of the theory of CBs in the context of IIGB and empirically verify the theoretical model underpinning our scale development; 2) to serve as an instrumental method for establishing the face validity and examining the predictive validity of the developed scale. Our preceding research, utilizing the IN-GBA as described, successfully accomplished the first objective [16], and the current investigation is using the IN-GBA to fulfil the second objective while developing the scale.

### 3.3 Dimension determination

Building upon the success the IN-GBA in validating the explanatory power of the CB theory in IIGB, and referring to the CHB scale developed by Knäuper et al. [9], which delineates dimensions based on various types of high-risk health behaviors such as smoking, alcohol consumption, and unhealthy dietary practices, we preliminarily established four distinct CB dimensions in the context of IIGB. These correspond to different types of IIGBs: online gaming compensation, binge-watching compensation, online shopping compensation, and other forms of compensation. These four categories represent the diverse preferences exhibited by instant gratifiers and acknowledge the possibility that a single individual may engage in a range of different types of IIGBs.

### 3.4 Item development

Informed by the CB theory [[7], [8], [9]] and enriched by the qualitative data harvested from our interviews and the IN-GBA, a preliminary 17-item scale, termed CBS-IIGB, was devised (see Appendix 1). The scale employs a seven-point Likert system, with scores ranging from 1 (strongly disagree) to 7 (strongly agree). Higher scores indicate stronger compensatory beliefs towards online instant gratification behaviors.

To ensure content validity, the generated items for the CBS-IIGB scale were reviewed by a panel of experts in behavioral psychology and online behavior. Experts were asked to rate each item's relevance to compensatory beliefs on a scale from 1 (not relevant) to 5 (highly relevant). Items that received an average rating below 3 were revised or discarded. This expert review ensured that the scale items were both comprehensively and representatively sampling the construct of interest.

### 3.5 Criterion measures

The IIGB is related to a lack of self-control, and frequent acquisition of internet instant gratification can lead to a tendency to form internet addiction. The prevalence of mobile internet has closely associated internet addiction with smartphone addiction [23,[25], [26], [27]]. Therefore, this study chose internet addiction, smartphone addiction, and self-control scales as criterion measures. Smartphone addiction includes a scale for college student smartphone addiction and adult smartphone addiction, which are used as criterion measures for the student and employee versions of the CBS-IIGB, respectively.

Chinese Internet Addiction Scale - Revised (CIAS-R) [35]: This scale consists of 19 items, encompassing four dimensions - internet addiction tolerance, compulsive internet use and internet addiction withdrawal response, time management issues, and interpersonal and health problems. Scores are on a four-point scale, ranging from 1 (strongly disagree) to 4 (strongly agree). Higher scores indicate a greater possibility and tendency towards internet addiction. A total score of less than 46 is considered normal; scores between 46 and less than 53 indicate internet dependency; scores of 53 or more indicate internet addiction. In this study, the Cronbach's α coefficient of each dimension among the employee sample was 0.79, 0.85, 0.83, 0.81, respectively; and in the student sample, it was 0.74, 0.80, 0.79, 0.79, respectively.

Smartphone Addiction Scale for College Students (SAS-CS) [36]: This scale includes 22 items and encompasses six factors - withdrawal reaction, salience, negative effects, social comfort, app use, and app updates. Scores are on a five-point scale, ranging from 1 (strongly disagree) to 5 (strongly agree). In this study, the Cronbach's α coefficients of the six dimensions were 0.68, 0.65, 0.81, 0.83, 0.76, and 0.51, respectively.

Smartphone Addiction Scale for Chinese Adults (SAS-CA) [37]: This scale contains 26 items and six factors - app use, app updates, withdrawal reaction, salience, social impairment, and physical discomfort. Scores are on a five-point scale, ranging from 1 (strongly disagree) to 5 (strongly agree). Higher scores indicate a more severe tendency towards smartphone addiction. In this study, the Cronbach's α coefficients of the six dimensions were 0.85, 0.75, 0.87, 0.77, 0.84, and 0.80, respectively.

Self-Control Scale for Chinese College Students (SCS-CCS) [38]: This scale comprises 19 items, including impulse control, healthy habits, resistance to temptation, focus on work, and restrained entertainment. Scores are on a five-point scale, ranging from 1 (strongly disagree) to 5 (strongly agree). Higher scores indicate a stronger trait self-control ability. In this study, the Cronbach's α coefficients of the five dimensions were 0.61, 0.62, 0.65, 0.73, and 0.85 for the employee sample; and 0.73, 0.63, 0.63, 0.56, and 0.52 for the student sample, respectively.

### 3.6 Statistical methods

We used SPSS 22.0 and Mplus7.0 for data processing and statistical analysis. Two independent sample t-tests and Pearson's correlation analysis between items were used to assess item discrimination. Exploratory Factor Analysis (EFA) and Confirmatory Factor Analysis (CFA) were used to evaluate the structural validity of the scale. Pearson's correlation was used to examine the relationship between the CBS-IIGB and criterion variables. Two independent sample t-tests were used to determine the differences in total CBS-IIGB scores between different behavioral choice groups. Cronbach's α coefficient test was used to test the internal consistency reliability for the total sample of both the employee and student versions. Pearson's correlation was used to test the test-retest reliability for a sample of college students (n = 100) and employees (n = 100).

## 4 Results

### 4.1 Item analysis

Upon reviewing the items with our expert panel, we found that the majority of items were rated as highly relevant to the construct of compensatory beliefs. The average relevance rating across all items was 4.7 out of 5. This reinforces the content validity of our scale, suggesting it accurately captures the nuances of compensatory beliefs in the context of online temptations.

Upon analyzing the item scores through independent sample t-tests between the top and bottom 27 % scorers (designated as high-scoring and low-scoring groups), significant differences were observed across all items (Item 8, *p* = 0.004; others, *p* < 0.001) except for item 11, leading to its exclusion. Pearson correlation analysis was conducted between individual item scores and the total score, and items with correlations below 0.4 were eliminated. This led to the removal of item 8. The retained items displayed correlations between 0.42 and 0.66 (*p* < 0.001 for all), affirming the items' internal consistency. The item analysis thus culminated with 15 items remaining.

### 4.2 Construct validity

#### 4.2.1 Exploratory factor analysis (EFA)

**Fig. 2:**
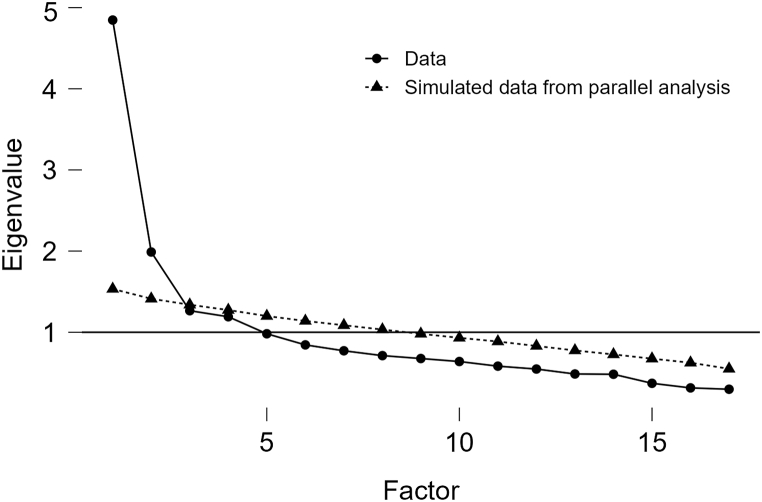
Scree plot for 1st round EFA (CBS-IIGB).

The suitability of data for factor analysis was affirmed by a KMO sample adequacy test (KMO = 0.827) and Bartlett's sphericity test (*χ*^*2*^ = 959.66, *df* = 199, *p* < 0.001). Principal component analysis with varimax rotation extracted four factors with eigenvalues over 1. However, the factor loadings of items on the initially confirmed four dimensions did not correspond one-to-one with factors, indicating that multiple items did not match the preset dimensions. Considering that the compensatory health beliefs scale developed by Knäuper et al. [9] was designed for a Canadian sample, and due to differences in thinking styles between Western and Chinese cultures [39], the dimensions established in reference to this scale may not yield the expected results in a Chinese cultural context. Therefore, items that did not meet the standard in exploratory analysis were eliminated for further research. To further validate our decision regarding the number of factors to retain, a scree plot was generated (Fig. 2). The plot displayed a pronounced inflection point after the second factor, suggesting a two-factor solution for our scale. This visual confirmation from the scree plot, combined with our eigenvalue criterion, provided strong support for a two-factor model despite the initial extraction of four factors based on eigenvalues. According to the criteria suggested by previous research, items were deleted if they met the following conditions: (1) factor loading <0.40; (2) absolute value of the maximum two cross-loadings ≥0.40; (3) cross-loadings were too close, i.e., the difference in the absolute values of the maximum two cross-loadings <0.10. Items 7, 13, and 17 had communalities lower than 0.4, and the absolute value of the maximum two cross-loadings of item 3 was >0.4, thus they were deleted. Item 2 had loadings lower than 0.4 on all four factors, and item 10 had cross-loadings, but it passed the item analysis. In order to ensure high reliability of the final scale, we cautiously screened the items in the prediction phase. After inspection, items 2 and 10 were found to be ambiguous, hence they were modified to align closely with the dimension meanings. The remaining items were examined and any that could lead to double loading in the EFA due to ambiguity were modified. The scale was then tested a second time.

The second test of the scale was divided into an employee version (CBS-IIGB-E) and a student version (CBS-IIGB-S). In the employee version, references to “studying” were changed to “working” or “overtime,” such as “Working overtime on the weekend can compensate for the time wasted on internet entertainment from Monday to Friday.” This was done to make the tool more accurate and targeted. After the first test, 11 items remained (see Appendix 2).

**Fig. 3:**
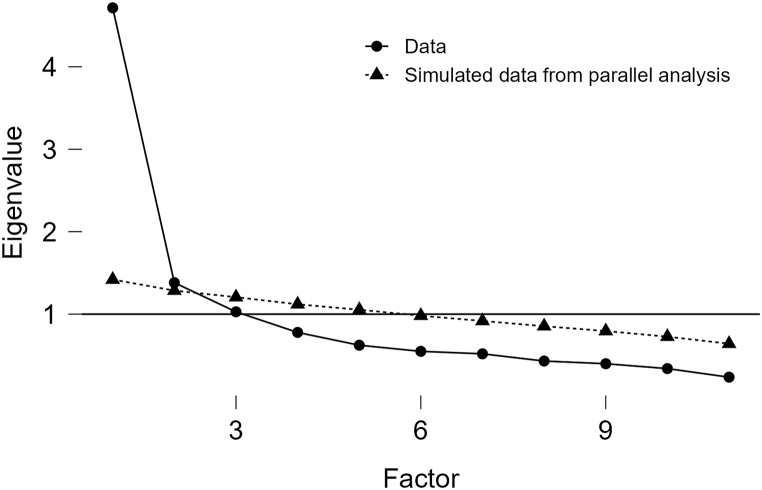
Scree plot for 2nd round EFA (CBS-IIGB-E).

For the second test of the employee version, we conducted item analysis and then an EFA. Item 1, which still had cross-loadings all greater than 0.4, was eliminated from the employee to include all three immediate gratification scenarios of online gaming, binge-watching, and online shopping, we changed “playing games” in item 2 to “binge-watching” and conducted a third test. Using principal component analysis, we extracted two factors with eigenvalues >1 using orthogonal rotation, which together explained 66.15 % of the variance. The first factor was named “Compensatory Beliefs for Working” and the second “Compensatory Beliefs for Resting”. Factor loadings are shown in Table 2a with descriptive statistics for each item shown in Table 2b. A scree plot (Fig. 3) was utilized to further validate the number of factors to be retained. The plot exhibited a clear inflection point after the second factor, supporting our decision to retain two factors for our scale. This visual assessment, in conjunction with the eigenvalues >1 criterion, provided consistent evidence for the two-factor solution of our scale.

**Table 2a:** Factor loadings of each item in CBS-IIGB-E

	Item	Loadings	
		Factor 1	Factor 2
Compensatory Beliefs for Working	1. If browsing shopping websites occupies work time, it can be made up by working a few more hours later.	**0.79**	0.25
	2. Working for an extra period can reduce the negative impact of previously binge-watching shows.	**0.72**	0.13
	3. Spending time on shopping website activities during work can be balanced by working extra time afterwards.	**0.75**	0.28
	4. Not playing games on workdays can compensate for the time spent excessive gaming last weekend.	**0.64**	0.21
	5. Working overtime on weekends can make up for the time wasted on online entertainment from Monday to Friday.	**0.73**	0.24
	6. Working a few more hours can compensate for the previous work time occupied by browsing shopping websites.	**0.84**	0.24
Compensatory Beliefs for Resting	7. If you stay up late watching shows at night, it is acceptable to make up for it by sleeping longer during the day.	0.24	**0.88**
	8. If you stay up late playing games at night, then sleep more the next day to make up for it.	0.21	**0.88**
	9. If you stay up late for the Double Eleven shopping spree, sleep more the next day to make up for it.	0.35	**0.74**
	10. Sleeping longer on weekends can compensate for the resting time occupied by playing with your phone on weekdays.	0.16	**0.76**

**Table 2b:** Descriptive statistics of each item in CBS-IIGB-E

Indicators	Item 1	Item 2	Item 3	Item 4	Item 5	Item 6	Item 7	Item 8	Item 9	Item 10
Mean	4.031	4.359	3.964	4.431	3.764	4.154	3.374	3.323	3.749	4.046
Std. Deviation	1.668	1.715	1.670	1.699	1.734	1.701	1.804	1.854	1.868	1.851
Skewness	−0.049	−0.349	−0.144	−0.377	0.069	−0.275	0.342	0.401	0.059	−0.078
Std. Error of Skewness	0.174	0.174	0.174	0.174	0.174	0.174	0.174	0.174	0.174	0.174
Kurtosis	−0.961	−0.903	−1.017	−0.931	−1.065	−0.983	−0.987	−1.051	−1.226	−1.187
Std. Error of Kurtosis	0.346	0.346	0.346	0.346	0.346	0.346	0.346	0.346	0.346	0.346

**Fig. 4:**
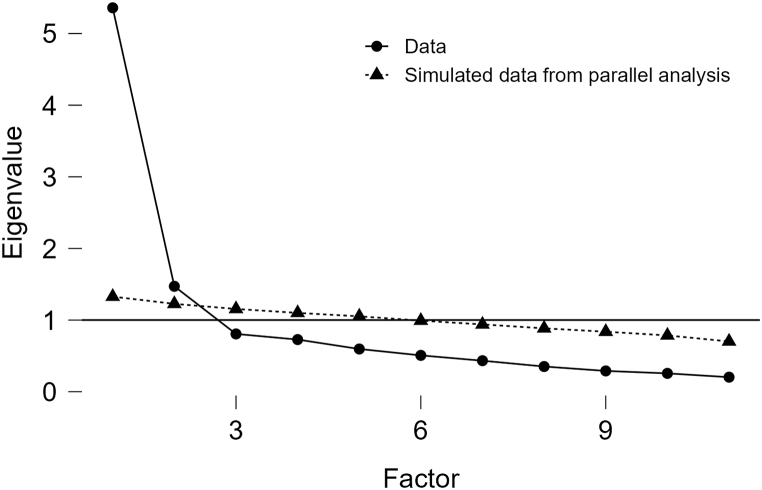
Scree plot for 2nd round EFA (CBS-IIGB-S).

We also performed item analysis and then an EFA on the results of the second test of the student version of the scale. Following the same criteria as with the employee version, we removed item 1 with communalities <0.4 and items 5 and 7 which still had cross-loadings all greater than 0.4. After re-analysis, we used principal component analysis and orthogonal rotation to extract two factors with eigenvalues >1. These factors explained 67.49 % of the variance. The first factor was named “Compensatory Beliefs for Studying” and the second “Compensatory Beliefs for Resting”. Factor loadings are shown in Table 3a with descriptive statistics for each item shown in Table 3b. Similarly, a scree plot (Fig. 4) was utilized to further validate the number of factors to be retained. The plot exhibited a clear inflection point after the second factor, supporting our decision to retain two factors for our scale. This visual assessment, in conjunction with the eigenvalues >1 criterion, provided consistent evidence for the two-factor solution of our scale.

**Table 3a:** Factor loadings of each item in CBS-IIGB-S.

	Item	Loadings	
		Factor 1	Factor 2
Compensatory Beliefs for Studying	1. Not watching shows on weekdays can make up for the time spent excessively binge-watching last weekend.	**0.71**	0.12
	2. Studying for a few more hours can compensate for the previous study time occupied by browsing shopping websites.	**0.84**	0.15
	3. Studying more on weekends can compensate for the time wasted on online entertainment from Monday to Friday.	**0.71**	0.30
	4. Studying for an extra period can reduce the negative impact of previous gaming.	**0.65**	0.13
Compensatory Beliefs for Resting	5. If you stay up late watching shows at night, it's acceptable to compensate by sleeping longer during the day.	0.14	**0.90**
	6. If you stay up late for the Double Eleven shopping spree, sleep more the next day to make up for it.	0.20	**0.82**
	7. If you stay up late playing games at night, then sleep more the next day to make up for it.	0.26	**0.85**
	8. Taking extra rest during the day can compensate for the sleep time occupied by staying up late online the previous night.	0.23	**0.86**

**Table 3b:** Descriptive statistics of each item in CBS-IIGB-S.

Indicators	Item 1	Item 2	Item 3	Item 4	Item 5	Item 6	Item 7	Item 8
Mean	4.425	4.800	4.390	5.000	3.490	3.735	3.255	3.650
Std. Deviation	1.590	1.504	1.695	1.407	1.671	1.667	1.736	1.668
Skewness	−0.343	−0.559	−0.297	−0.601	0.125	0.053	0.503	0.109
Std. Error of Skewness	0.172	0.172	0.172	0.172	0.172	0.172	0.172	0.172
Kurtosis	−0.755	−0.285	−0.814	−0.195	−1.049	−1.106	−0.801	−1.056
Std. Error of Kurtosis	0.342	0.342	0.342	0.342	0.342	0.342	0.342	0.342

#### 4.2.2 Confirmatory factor analysis (CFA)

We conducted a CFA on the final 10-item employee version to examine the fit between the conceived two-factor model and the actual model. The sample size met general recommendations for CFA, providing sufficient power to detect medium-sized effects. Our post-hoc power analysis indicated that the power for our primary CFA was approximately 0.93, suggesting a robust ability to detect the specified model fit. We employed the robust maximum likelihood method (MLM) for the CFA, comparing various fit indices. Based on the recommendations from McNeish and Wolf (2022) [40], we adjusted our fit index cutoffs for our two-factor model. The fit results showed the model fit indices were: *χ*^*2*^/*df* = 2.061, CFI = 0.960 (noting that values as low as 0.90 might be acceptable for one-factor models, making our value of 0.960 very satisfactory), TLI = 0.947, RMSEA = 0.079 (with the adjusted recommendation suggesting values closer to 0.05, our value is marginally higher but still within a reasonable range), SRMR = 0.053 (consistent with the conventional cutoff of 0.08). Overall, these indices indicate a satisfactory fit, especially when considering the dynamic cutoffs for one-factor models. The final version of the scale is presented in Appendix 3.

Similarly, we conducted a CFA on the final 8-item student version using the maximum likelihood method to examine the fit between the conceived two-factor model and the actual model. The fit results showcased good model fit: *χ*^*2*^/*df* = 1.014, CFI = 0.999, TLI = 0.999, RMSEA = 0.008 (well within the adjusted recommended value), SRMR = 0.033. These fit statistics further affirm the suitability of our two-factor model for the student version. The final version of this scale is likewise presented in Appendix 3.

#### 4.2.3 Criterion validity

As shown in Table 4, the CBS-IIGB-E overall score and its dimension scores showed significant positive correlations with the CIAS-R and SAS-CA total scores and their dimension scores, and significant negative correlations with the SCS-CCS dimension scores.

**Table 4:** Correlation (r) between each dimension of the CBS-IIGB-E scale and criterion measures.

		CBs Total Score	CBs for Working	CBs for Resting
CIAS-R	CIAS-R Internet Addiction Total Score	0.32***	0.31***	0.25***
	Time Management Disorder	0.30***	0.32***	0.20**
	Internet Addiction Tolerance	0.25***	0.23***	0.22**
	Compulsive Internet Use and Internet Addiction Withdrawal	0.29***	0.26**	0.26***
	Interpersonal and Health Issues	0.26***	0.27***	0.18**
SAS-CA	SAS-CA Total Score	0.33***	0.33***	0.25***
	App Use	0.28***	0.27***	0.22**
	App Update	0.30***	0.31***	0.20**
	Withdrawal Reaction	0.29***	0.25***	0.27***
	Salience	0.28***	0.27***	0.27***
	Social Function Impairment	0.26***	0.28***	0.17*
	Physical Discomfort	0.26***	0.29***	0.16*
SCS-CCS	SCS-CCS Total Score	−0.36***	−0.31***	−0.33***
	Resisting Temptation	−0.19**	−0.15*	−0.18*
	Focus on Work	−0.30***	−0.23**	−0.30***
	Healthy Habits	−0.34***	−0.26***	−0.35***
	Entertainment Containment	−0.29***	−0.25***	−0.26***
	Impulse Control	−0.35***	−0.34***	−0.28***

Note：***.*p* < 0.001 ，**p < 0.01 ，*p < 0.05.

As shown in Table 5, the CBS-IIGB-S overall score had significant positive correlations with the CIAS-R, SAS-CS total scores, and their dimension scores, and significant negative correlations with the SCS-CCS and its dimensions. The “Learning Compensation” dimension had significant positive correlations with the CIAS-R total score, tolerance of internet addiction, compulsive internet use, and the withdrawal reaction of internet addiction. It was not significantly correlated with the other two dimensions. It showed a significant positive correlation with the SAS-CS total score and its emergent behavior, negative influence, withdrawal behavior, and social soothing dimensions, but was not significantly related to the app update and app use dimensions. It had a significant correlation with the SCS-CCS focus on work dimension score, but was not significantly related to the SCS-CCS total score and the rest of its dimensions. “Rest-Activity Compensation” had a significant positive correlation with CIAS-R, SAS-CS total scores, and all their dimensions and showed significant negative correlations with the SCS-CCS total score and its dimensions.

**Table 5:** Correlation (r) between each dimension of the CBS-IIGB-S scale and criterion measures.

		CBs Total Score	CBs for Studying	CBs for Resting
CIAS-R	CIAS-R Total Score	0.25***	0.16**	0.26***
	Time Management Disorder	0.18**	0.11	0.20**
	Internet Addiction Tolerance	0.21***	0.14*	0.20***
	Compulsive Internet Use and Internet Addiction Withdrawal	0.31***	0.20***	0.31***
	Interpersonal and Health Issues	0.14*	0.07	0.15**
SAS-CS	SAS-CS Total Score	0.29***	0.19**	0.29***
	Striking Behavior	0.22***	0.12*	0.25***
	Negative Impact	0.22***	0.18**	0.19***
	Withdrawal Behavior	0.25***	0.17**	0.25***
	App Update	0.14*	0.11	0.13*
	App Use	0.22***	0.09	0.27***
	Social Soothing	0.19**	0.13*	0.18*
SCS-CCS	SCS-CCS Total Score	−0.21***	−0.10	−0.24***
	Resisting Temptation	−0.14*	−0.04	−0.18**
	Healthy Habits	−0.22***	−0.10	−0.26***
	Impulse Control	−0.12*	−0.05	−0.13*
	Focus on Work	−0.17**	−0.15*	−0.14*
	Entertainment Containment	−0.18**	−0.08	−0.21***

Note：***p < 0.001，**.*p* < 0.01 ，*p < 0.05.

#### 4.2.4 Predictive validity

We used an online instant gratification behavior simulation questionnaire to test whether the CBS-IIGB could accurately predict compensatory beliefs in online instant gratification behavior. We coded the results based on whether the temptation of online instant gratification was accepted (accepted as “1″, rejected as “0″), whether the compensatory belief was activated (directly accepted or rejected temptation as “0″, activated compensatory belief as “1″), and whether the compensatory behavior was carried out after activating the compensatory belief (not carried out as “0″, carried out as “1″). This questionnaire was presented to the participants after they answered demographic questions and underwent the CBS-IIGB assessment. The full content can be found in the link provided in Appendix 4.

The results showed that in the employee group, using the independent samples *t*-test to compare the CBS-IIGB-E total scores of participants making different choices in the simulation questionnaire, the results are shown in Table 6. The comparison based on whether the temptation of online instant gratification was accepted was statistically significant (*t* = 6.46, *p* < 0.001). The comparison based on whether the compensatory belief was activated was statistically significant (*t* = 5.13, *p* < 0.001). The comparison based on whether the compensatory behavior was carried out after activating the compensatory belief showed no significant difference (*t* = 0.66, *p* = 0.53).

**Table 6:** Comparison of CBS-IIGB-E scores among different choices in the context of internet instant gratification scenarios.

Comparison Category	*N*	$\bar{x}$ ± *s*	*t*	*p*
Temptation Accepted (including direct temptation acceptors and compensatory belief activators)	116	43.64 ± 11.06	6.46	<0.001
Temptation Resisted	79	32.87 ± 11.65		
Compensation Belief Not Activated (including direct temptation acceptors/resisters)	109	35.49 ± 12.19	−5.13	<0.001
Compensation Belief Activated	86	44.08 ± 11.13		
Compensation Belief Activated, Temptation Accepted but Compensation Behavior Not Executed	27	45.26 ± 12.43	0.66	0.53
Compensation Belief Activated and Compensation Behavior Executed	59	43.54 ± 10.56		

In the student group, using the independent samples *t*-test to compare the CBS-IIGB-S total scores of participants making different choices in the simulation questionnaire, the results are shown in Table 7. The comparison based on whether the temptation of online instant gratification was accepted was statistically significant (*t* = 3.04, *p* = 0.003). The comparison based on whether the compensatory belief was activated was statistically significant (*t* = 3.45, *p* < 0.001). The comparison based on whether the compensatory behavior was carried out after activating the compensatory belief showed no significant difference (*t* = 0.65, *p* = 0.52).

**Table 7:** Comparison of CBS-IIGB-S scores among different choices in the context of internet instant gratification scenarios.

Comparison Category	*N*	$\bar{x}$ ± *s*	*t*	*p*
Temptation Accepted (including direct temptation acceptors and compensatory belief activators)	132	34.12 ± 7.81	3.04	0.003
Temptation Resisted	68	30.07 ± 9.43		
Compensation Belief Not Activated (including direct temptation acceptors/resisters)	127	31.31 ± 9.18	−3.45	<0.001
Compensation Belief Activated	73	35.24 ± 6.82		
Compensation Belief Activated, Temptation Accepted but Compensation Behavior Not Executed	21	35.70 ± 5.98	0.65	0.52
Compensation Belief Activated and Compensation Behavior Executed	52	34.94 ± 7.17		

These results indicate that the CBS-IIGB can accurately predict individual compensatory beliefs in online instant gratification behaviors, i.e., individuals with higher CBS-IIGB scores are more likely to activate compensatory beliefs and accept temptations, but it does not predict whether they will carry out the compensatory behavior.

#### 4.2.5 Reliability analysis

The Cronbach's α coefficients of the CBS-IIGB-E overall scale and its two dimensions were 0.87, 0.82, and 0.85, respectively; the CBS-IIGB-S overall scale and its two dimensions' Cronbach's α coefficients were 0.82, 0.77, and 0.86. Furthermore, to assess the reliability of the CBS-IIGB scale in a multidimensional context, we utilized McDonald's Omega (ω) as a measure of internal consistency. This metric is especially appropriate for scales with multiple factors, as it considers the variance of each factor and the covariance between factors, providing a more accurate estimate than traditional measures such as Cronbach's alpha.

For the CBS-IIGB-E (employee version): The ω coefficient for the overall scale was 0.88. For the “Compensatory Beliefs for Working” dimension, ω was 0.84. For the “Compensatory Beliefs for Resting” dimension, ω was 0.86.

For the CBS-IIGB-S (student version): The ω coefficient for the overall scale was 0.85. For the “Compensatory Beliefs for Studying” dimension, ω was 0.82. For the “Compensatory Beliefs for Resting” dimension, ω was 0.87.

The test-retest reliability (r) of the CBS-IIGB-E overall scale was 0.76 (p < 0.001), and the test-retest reliability of its two dimensions was 0.64 and 0.72 (p < 0.001). The test-retest reliability of the CBS-IIGB-S overall scale was 0.76 (p < 0.001), and the test-retest reliability of its two dimensions was 0.64 and 0.75 (p < 0.001).

## 5 Discussion

In today's digital age, the allure of instant internet gratification poses significant challenges to self-regulation, especially among the youth [41]. Our research aimed to operationalize the measurement of compensatory beliefs within the realm of online instant gratification and offer insights for preemptive interventions in mental health practices. We meticulously crafted and validated two distinct versions of the CBS-IIGB, one tailored for organizational employees and another for college students, addressing the unique nuances and challenges inherent to each demographic.

### 5.1 Theoretical underpinnings and cultural considerations

Our findings substantiate that the CBS-IIGB, in its two distinct versions, emerged as a robust instrument with dual dimensions reflecting the multifaceted nature of compensatory beliefs. Both exploratory and confirmatory factor analyses bolstered the structure of the scale. These dimensions encapsulate diverse strategies individuals employ to reconcile the tension between succumbing to digital temptations and fulfilling their roles or commitments.

At its core, this tension can be viewed through the lens of temporal discounting [42], where individuals heavily favor immediate rewards, such as internet gratifications, over more significant future rewards like completing a work assignment or studying for an exam. Such a perspective of valuing the present more than the future can be further exacerbated when individuals are mentally fatigued, as posited by the ego depletion theory [43]. The finite nature of self-control means that after resisting numerous daily temptations, individuals might resort to compensatory beliefs to rationalize their digital indulgences. Moreover, the discomfort arising from acting against one's values, such as wasting time online, can be understood using the cognitive dissonance theory [12]. To reduce this dissonance, individuals might develop compensatory beliefs, serving as self-created justifications for their behavior. In the realm of behavioral psychology, operant conditioning can shed light on the allure of the internet. The immediate rewards from internet use, such as likes or notifications, act as positive reinforcers, strengthening the behavior of frequent checking and browsing. Compensatory beliefs might serve as cognitive strategies to manage potential negative outcomes from such conditioned behaviors.

In light of divergent thinking styles influenced by cultural contexts [39], our study echoes the necessity for culturally consonant measurement tools. The initial incongruence of item loadings, when construed in reference to a Western-developed questionnaire, underscores the importance of cultural tailoring. By recalibrating the items to resonate with holistic thinking patterns prevalent in Chinese culture, we refined the scale to better fit our cohorts. This cultural emphasis is crucial, especially when considering previous studies on compensatory beliefs in domains like diet or exercise, which may have been influenced by cultural contexts different from internet gratification behaviors. Further study could validate our instrument in other cultures and see if our calibration holds.

Beyond the evident cultural differences, our findings invite a deeper contemplation on the universality and variability of compensatory beliefs across cultures. While the overarching concept of compensatory beliefs is not new, its manifestation in the realm of digital behaviors presents a novel frontier for theoretical exploration. Do these beliefs function as cognitive mechanisms to balance immediate gratifications with long-term goals universally, or are they more pronounced in certain cultural or demographic groups? Our study suggests that while the essence of these beliefs might be universal, their articulation and intensity could be shaped by cultural nuances. This interplay between individual cognition and collective culture necessitates a holistic theoretical framework that integrates individual psychology, cultural anthropology, and digital sociology.

### 5.2 Practical implications for targeted interventions

The bifurcation of the CBS-IIGB into student and employee versions underscores its practical potential. By catering to the unique contexts of college students and organizational employees, this instrument offers granular insights, enabling mental health practitioners to devise tailored interventions. Recognizing the consequences of unfulfilled compensatory behaviors can guide strategies to disrupt the cycle, potentially mitigating the onset of internet addiction and associated mental health challenges like depression [16]. Therefore, mental health workers can take inspiration from this instrument, pay attention to this at-risk group, provide preemptive intervention, and thus reduce the incidence of internet addiction.

Regarding the naming of the two dimensions, we considered that being affected by network temptation reduces learning efficiency, and “cyberloafing” in work [44,45] are common in daily life. This not only hinders personal development but also wastes resources and affects organizational performance. Therefore, the first dimensions of the CBS-IIGB student version and the employee version are named “Compensatory Beliefs for Studying” and “Compensatory Beliefs for Working " respectively, indicating to what extent the participants agree that increasing study or work time can compensate for the negative impact of online instant gratification. On the other hand, as mobile networks become more common and smartphones become increasingly convenient, people's bedtime is getting later [46,47]. This situation exists in both the employee and student groups, so the second dimensions of both versions are named “Compensatory Beliefs for Resting”. The content of “Compensatory Beliefs for Resting” indicates the extent to which the participants agree that compensatory sleep can compensate for the negative impact of staying up late on the internet. These dimensions emphasize the relevance of our scale in addressing the multifaceted repercussions of digital immersion.

### 5.3 Expanding the scope and future directions

While our study has tailored the CBS-IIGB for students and employees, the applicability of these scales to broader populations remains to be validated. Future research endeavors could endeavor to adapt the CBS-IIGB to various demographic segments, thereby enhancing its utility across diverse societal strata. Additionally, theoretical debates could benefit from dissecting the psychological constructs underlying compensatory beliefs and their manifestation in behavior. Further research might explore the neurological correlates of compensatory beliefs and their behavioral outcomes, particularly in the context of digital engagement. Such investigations could bridge the gap between the theoretical underpinnings of compensatory beliefs and their practical implications, potentially informing both preventative and remedial interventions in the digital age.

## 6 Concluding thoughts

By crafting a culturally attuned and empirically validated scale, this study contributes a significant tool to the psychology of internet use. It lays the groundwork for both theoretical advancement and practical application in understanding and managing the complex interplay between instant digital gratification and compensatory beliefs.

## Declarations

The studies involving human participants were reviewed and approved by the Institutional Review Board of School of Psychology, Fujian Normal University (Approval No. 2020090402). The participants provided their written informed consent to participate in this study.

## CRediT authorship contribution statement

**Bin Yin:** Conceptualization, Data curation, Formal analysis, Funding acquisition, Investigation, Methodology, Project administration, Resources, Supervision, Validation, Writing – original draft, Writing – review & editing. **Yong Shen:** Conceptualization, Data curation, Formal analysis, Investigation, Methodology, Validation, Writing – original draft.

## Declaration of competing interest

The authors declare that they have no known competing financial interests or personal relationships that could have appeared to influence the work reported in this paper.

## Appendix

### Appendix 1 First Assessment of CBS-IIGB

1. Playing games in the morning can be balanced by studying for an extended period in the afternoon or evening.
2. Abstaining from gaming from Monday to Friday can compensate for the time consumed by excessive gaming over the weekend.
3. Frequently gaming can be counteracted by concentrating more during study time and refraining from looking at your mobile phone.
4. If you stay up late gaming at night, it's acceptable to compensate by sleeping longer during the day.
5. Studying for a few extra hours can make up for the study time previously occupied by gaming.
6. The time spent choosing items on Taobao can be offset by studying for a few additional hours.
7. The original study time occupied by engaging in Double 11 event tasks can be compensated by the cash rewards earned from the event.
8. The stress brought about by studying can be alleviated through the pleasure derived from shopping.
9. If you stay up late shopping during Double 11, you can compensate by sleeping longer the next day.
10. If you waste a lot of time scrolling through Taobao today, you can make up for it by studying diligently tomorrow.
11. Watching TV shows for relaxation can alleviate the pressure brought about by a whole day's work.
12. Browsing short videos during study time can serve as a relaxation method.
13. If you haven't followed any series for a week, you can catch up on the episodes you missed when they are updated next week.
14. Regularly watching TV series is acceptable if all study tasks can be completed on time.
15. High levels of stress can be compensated by watching TV series.
16. Studying more over the weekend can compensate for the time wasted from Monday to Friday.
17. Resting over the weekend can compensate for the pressure experienced from studying during the week.

## Appendix 2 Second Assessment of CBS-IIGB

### Employee Version

1. If watching shows in the evening disrupts work, it's acceptable to compensate by working overtime at night.
2. If browsing shopping websites takes up work time, working a few extra hours later can make up for it.
3. Working for an extended period can mitigate the negative impact of previous gaming activities.
4. Spending work time on shopping website tasks can be balanced by working extra hours afterwards.
5. Refraining from gaming on workdays can compensate for the time consumed by excessive gaming over the last weekend.
6. Working overtime on the weekend can make up for time wasted on online entertainment from Monday to Friday.
7. Working a few extra hours can offset the time previously spent browsing shopping websites.
8. If you stay up late watching shows at night, it's acceptable to compensate by sleeping longer during the day.
9. If you stay up late gaming at night, you can make up for it by sleeping longer the next day.
10. If you stay up late shopping during Double 11, you can compensate by sleeping longer the next day.
11. Sleeping longer over the weekend can compensate for the rest time occupied by using the phone during regular days.

### Student Version

1. If a friend invites me to play games, I can join and then make up for it by studying for an extended period later.
2. Not watching shows on workdays can compensate for the time consumed by excessive binge-watching over the last weekend.
3. Studying for a few extra hours can compensate for the study time previously spent browsing shopping websites.
4. Studying more over the weekend can make up for the time wasted on online entertainment from Monday to Friday.
5. If gaming takes up study time, studying for a few extra hours later can make up for it.
6. Studying for an additional period can mitigate the negative impact of previous gaming activities.
7. Choosing to play games during self-study time can be balanced by studying for an extended period afterwards.
8. If you stay up late watching shows at night, it's acceptable to compensate by sleeping longer during the day.
9. If you stay up late shopping during Double 11, you can compensate by sleeping longer the next day.
10. If you stay up late gaming at night, you can make up for it by sleeping longer the next day.
11. Resting more during daytime free time can compensate for the sleep time occupied by staying up late online the previous night.

## Appendix 3 Third Assessment of CBS-IIGB

Employee Version.

1. If browsing shopping websites occupies work time, it can be made up by working a few more hours later.
2. Working for an extra period can reduce the negative impact of previously binge-watching shows.
3. Spending time on shopping website activities during work can be balanced by working extra time afterwards.
4. Not playing games on workdays can compensate for the time spent excessive gaming last weekend.
5. Working overtime on weekends can make up for the time wasted on online entertainment from Monday to Friday.
6. Working a few more hours can compensate for the previous work time occupied by browsing shopping websites.
7. If you stay up late watching shows at night, it is acceptable to make up for it by sleeping longer during the day.
8. If you stay up late playing games at night, then sleep more the next day to make up for it.
9. If you stay up late for the Double Eleven shopping spree, sleep more the next day to make up for it.
10. Sleeping longer on weekends can compensate for the resting time occupied by playing with your phone on weekdays.

Student Version.

1. Not watching shows on weekdays can make up for the time spent excessively binge-watching last weekend.
2. Studying for a few more hours can compensate for the previous study time occupied by browsing shopping websites.
3. Studying more on weekends can compensate for the time wasted on online entertainment from Monday to Friday.
4. Studying for an extra period can reduce the negative impact of previous gaming.
5. If you stay up late watching shows at night, it's acceptable to compensate by sleeping longer during the day.
6. If you stay up late for the Double Eleven shopping spree, sleep more the next day to make up for it.
7. If you stay up late playing games at night, then sleep more the next day to make up for it.
8. Taking extra rest during the day can compensate for the sleep time occupied by staying up late online the previous night.

## Appendix 4 CBS-IIGB with IN-GBA (Chinese language)

Employee Version**：**https://www.credamo.com/answer.html#/s/VvQB32_

Student Version**：**https://www.credamo.com/answer.html#/s/JvMFni_

## Footnotes

1 A powerful, large-scale and reliable smart research platform that provides both online research design and data collection services in both the Chinese and English languages (https://www.credamo.com). It has a subject pool of more than 2.8 million participants and is nationally representative all across different age, gender, income, education and career fields.
